# Supplementary material for: MetaRibo-Seq measures translation in microbiomes
Source: Nat Commun. 2020 Jun 29;11:3268. doi: 10.1038/s41467-020-17081-z (PMC7324362; doi:10.1038/s41467-020-17081-z)
Supplement: Supplementary file 10 — Supplementary Data 7 [file 41467_2020_17081_MOESM10_ESM.zip › File2/Confidence_VeryHigh_Taxonomy/406382_out.krona.html]

Javascript must be enabled to view this page.

members
magnitude
magnitudeUnassigned
count
unassigned
taxon
rank

406382\_out

5

superkingdom
1
2157


SRS077016\_contig\_number\_19572
1
species
1579373


SRS149784\_contig\_number\_17745
1

3
superkingdom
2

1239
3
phylum

186801
3
class

3
order
186802

3
541000
family


SRS049959\_contig\_number\_32937SRS052697\_contig\_number\_contig-100\_3471.220076SRS142503\_contig\_number\_58679
3
1898205
species
